# Optical properties of graphene oxide

**DOI:** 10.3389/fchem.2023.1214072

**Published:** 2023-07-20

**Authors:** Talia Tene, Marco Guevara, Freddy Benalcázar Palacios, Tania Paulina Morocho Barrionuevo, Cristian Vacacela Gomez, Stefano Bellucci

**Affiliations:** ^1^ Department of Chemistry, Universidad Técnica Particular de Loja, Loja, Ecuador; ^2^ Facultad de Ingeniería Mecánica, Escuela Superior Politécnica de Chimborazo (ESPOCH), Riobamba, Ecuador; ^3^ Facultad de Ingeniería en Sistemas Electrónica e Industrial, Universidad Técnica de Ambato, Ambato, Ecuador; ^4^ Facultad de Ciencias, Carrera de Estadística, Escuela Superior Politécnica de Chimborazo (ESPOCH), Riobamba, Ecuador; ^5^ INFN-Laboratori Nazionali di Frascati, Frascati, Italy

**Keywords:** graphene oxide, optical bandgap, electronic transitions, absorption coefficient, Tauc analysis

## Abstract

The study of the optical properties of graphene oxide (GO) is crucial in designing functionalized GO materials with specific optical properties for various applications such as (bio) sensors, optoelectronics, and energy storage. The present work aims to investigate the electronic transitions, optical bandgap, and absorption coefficient of GO under different conditions. Specifically, the study examines the effects of drying times ranging from 0 to 120 h while maintaining a fixed temperature of 80°C and low temperatures ranging from 40 ℃ to 100 ℃ , with a constant drying time of 24 h. Our findings indicate that exposing the GO sample to a drying time of up to 120 h at 80°C can lead to a reduction in the optical bandgap, decreasing it from 4.09 to 2.76 eV. The $\pi -\pi^{*}$ transition was found to be the most affected, shifting from approximately 230 nm at 0 h to 244 nm after 120 h of drying time. Absorption coefficients of 3140–5507 ml mg^−1^ m^−1^ were measured, which are similar to those reported for exfoliated graphene dispersions but up to two times higher, confirming the improved optical properties of GO. Our findings can provide insights into determining the optimal temperature and duration required for transforming GO into its reduced form for a specific application through extrapolation. The study is complemented by analyzing the elemental composition, surface morphology change, and electrical properties.

## 1 Introduction

Over the past decade, oxidized graphenes have gained significant attention due to their unique physical and chemical properties (Chaudhuri and Yun, 2023). For instance, graphene oxide (GO) is produced by oxidizing graphene, a two-dimensional honeycomb material, with strong acids and oxidizing agents (Haydari et al., 2023). This process introduces oxygen-containing functional groups, such as hydroxyl, epoxy, and carboxyl groups (Ferrari et al., 2023), onto the graphene lattice and modifies its electronic structure, creating an intrinsic bandgap. GO offers several advantages over pure graphene, including improved processability, versatility, and cost-effectiveness (Grewal et al., 2018). Specifically, GO possesses interesting properties such as high hydrophilicity, solubility in water, large surface area, excellent dispersibility, and biocompatibility, making it an attractive material for various applications in fields such as energy storage (Safari and Mazloom, 2023), biosensors (Kadhim et al., 2023), water purification (Tene et al., 2022a), electronic devices (Wu et al., 2023), and drug delivery (Sontakke et al., 2023).

However, the presence of oxygen-containing functional groups in GO diminishes its electrical conductivity and mechanical strength (Liu et al., 2022). To address these limitations, GO is often reduced to obtain reduced GO (rGO), which restores the sp^2^ carbon network and partially recovers the electronic properties of graphene (Yu et al., 2023). This reduction process can be achieved chemically, electrochemically, or thermally. Among these, the thermal reduction at high temperatures ( >900℃ ) is the most attractive since it does not imply the use of strong chemical agents or subsequent processes. As a result, rGO has a lower oxygen content and higher electronic conductivity compared to GO while retaining some of the unique properties of graphene such as large surface area and excellent electrical conductivity.

The electronic and optical properties of GO and rGO are critical for next-generation devices, making it essential to comprehend their behavior in various environments. Explicitly, understanding the electronic transitions and bandgap of these materials is important for optimizing their performance in electronic and photonic devices, designing functional materials, and identifying potential applications. The bandgap of GO and rGO can be estimated using various techniques, including X-ray photoelectron spectroscopy (XPS) and UV-visible spectroscopy (Baragau et al., 2023; Nagaiah et al., 2023). In particular, the Tauc method, which is widely used for estimating the bandgap of semiconducting materials from the absorption spectrum, has also been applied to GO and rGO in several studies (Aragaw, 2020). This method assumes that the absorption coefficient follows a power law as a function of photon energy, and the bandgap can then be estimated as the intercept of the linear portion with the x-axis. Therefore, the Tauc approach can be a useful approximation for estimating the bandgap of oxidized graphenes.

To put the reason for this research field in a broader context, Kumar et al. (2014) introduced a scalable thermal annealing process to enhance the properties of graphene oxide (GO). The annealing induces a phase transformation, resulting in improved optical and electronic properties of GO without compromising its oxygen content. The findings offer a pathway for the bulk processing of GO with enhanced properties for various applications. Very recently, Valentini et al., (2023) investigated the use of low-temperature thermal annealing to tune the electrical properties of GO and rGO. The authors optimized the annealing conditions and show that it is possible to achieve low resistivities and enhanced electrochemical performance in rGO films. This approach is scalable, environmentally friendly, and holds promise for applications in flexible and wearable electronics. These papers propose intriguing methods for producing GO in large quantities with specified features, however, they do not provide further information about the optical bandgap, electrical transitions, or absorption coefficients. To fully understand the possibilities of this strategy in changing the properties of GO, more research is therefore required.

Additionally, in our previous study (Arias Arias et al., 2020), we investigated the effects of drying time on GO with a specific focus on a maximum drying time of 24 h at a temperature of 80°C. Through the use of Raman spectroscopy, UV-visible spectroscopy, and TEM, we discussed changes in defects, absorption spectra, as well as the stacking of GO sheets. The findings contribute to enhancing the production of GO powder, within the limitations of the drying time and temperature parameters explored. With this in mind, in the present work, we extended our study to widely understand the change in electronic transitions, optical bandgap, and spectral weight of several GO samples under the effects of drying time (up to 120 h) and low temperatures (80°C and 50°C). Our analysis includes UV-visible spectroscopy to estimate the bandgap using the Tauc method, as well as energy-dispersive X-ray spectroscopy (EDS) and scanning electron microscopy (SEM) to gain additional insights into the elemental composition and morphology of the obtained materials. Additionally, we discuss in detail the values of the optical absorption coefficient under different environments as well as the related electrical characteristics. These findings provide never-discussed valuable insights into the electronic transitions and optical properties of GO.

## 2 Materials and methods

**FIGURE 1:**
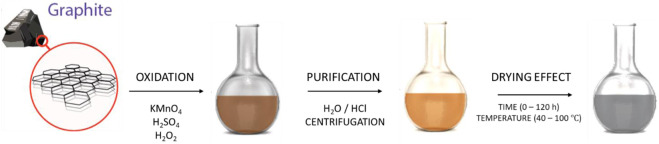
Schematic representation of the synthesis process.

It is important to note that details of the oxidation-reduction process and the successful transformation of GO into chemically treated rGO can be found in our previous works (Arias Arias et al., 2020; Tene et al., 2021), along with its applications in pollutant removal, such as methylene blue (Arias Arias et al., 2020) and Hg(II) (Tene et al., 2022b). Here, we provide a brief overview of the synthesis process (Figure 1) and concentrate on discussing the chemical and physical properties of thermally treated GO.

### 2.1 Materials

Graphite powder (150 m, 99.99%), sulfuric acid (H_2_SO_4_, ACS reagent, 95.0%–98.0%), potassium permanganate (KMnO_4_, ACS reagent, 99.0%), and hydrochloric acid (HCl, ACS reagent, 37%) were purchased from Sigma Aldrich. Hydrogen peroxide (H_2_O_2_, 30%) was obtained from Merk. All chemicals were used as received without further purification.

### 2.2 Synthesis of GO

A homogeneous mixture was obtained by adding 3.0 g of graphite powder to 70 mL of H_2_SO_4_ and stirring. The mixture was then placed in an ice bath, and 9 g of KMnO_4_ was added, keeping the temperature under 20°C. After 30 min, the mixture was transferred to a water bath and heated to 50°C for 30 min while constantly stirring. Subsequently, 150 ml of distilled water was gradually added to the solution over 20 min, ensuring that the temperature did not exceed 90°C. Then, 500 ml of distilled water was added, and 15 ml of H_2_O_2_ was introduced. After 1 h, the precipitated material was divided into centrifuge tubes and washed with a 1:10 solution of HCl and distilled water via several centrifugations at 10000 rpm for 10 min. The precipitate was then placed in a Teflon container and dried in an oven at a temperature of 45°C for 48 h to obtain graphite oxide powder.

For low-temperature treatment, 100 mg of graphite oxide powder was sonicated for 30 min in 500 ml of distilled water and centrifuged at 500 rpm for 10 min to obtain a homogeneous GO suspension. This suspension was divided into two equal parts. The first part was dried at 80°C considering different drying times ranging from 0 h to 120 h. The second part was dried considering different temperatures from 40°C to 100°C and fixing the drying time at 24 h. The temperature treatment was carried out in a POL-EKO drying stove.

After treatment (drying time or temperature), the samples were sonicated for several minutes to re-disperse them before measuring their optical properties. However, it should be noted that the samples kept at 120°C for 24 h and 80°C for 120 h required an extra sonication time compared to the other samples.

### 2.3 Characterization

The absorption spectra of GO and rGO were recorded using a Thermo Scientific Evolution 220 spectrophotometer with a resolution of 0.1 nm in a wavelength window from 190 to 1000 nm. The optical absorption coefficient was obtained by setting λ=660 nm. Quartz cuvettes (3.5 ml) with a 10 mm optical path were used. Spectra were normalized to the maximum of the prominent peak and conventional Lorentz functions were used to fit the curve. The surface morphology of the obtained samples was taken out on a scanning electron microscope (SEM, JSM-IT100 InTouchScope) with an accelerating voltage of 20 kV and equipped with a JEOL-made energy-dispersive X-ray spectrometer (EDS). The electrical characterization was carried out by using a KEI2450 instrument. Raman measurements were carried with a LabRAM HR Evolution micro-Raman spectrometer (Horiba Jobin-Yvon, operating at 532 nm).

## 3 Results and discussions

To provide context for the significance of our work, it is important to understand the various methods available for treating GO. These methods include chemical, electrochemical, and thermal reduction approaches.

• Chemical reduction involves using agents such as hydrazine, sodium borohydride, hydroiodic acid, or citric acid to remove oxygen functional groups, restoring the sp^2^ carbon network. While simple, this method may introduce defects and impurities (Tene et al., 2022b).
• Electrochemical reduction applies a voltage or current to a GO electrode immersed in a reducing electrolyte solution (Tian et al., 2023). It offers better control and higher-quality results but requires specialized equipment and time.
• Thermal reduction involves heating GO at high temperatures in the presence of a reducing agent, resulting in the breakdown of functional groups and the restoration of the graphene lattice. Different heating methods and reducing agents can be used (Sengupta et al., 2018). This approach is simple and cost-effective but requires high temperatures and long processing times.

Some works have explored low-temperature reduction or thermolysis, where GO is heated below 100°C in a vacuum or inert atmosphere (Wang et al., 2012). Despite numerous studies on GO reduction, there is a lack of reports on a simple, low-temperature treatment without a reducing agent or controlled atmosphere.

### 3.1 Absorption spectra *vs*. drying time

The temperature of 80°C was selected as a representative value within the studied temperature range. By keeping the temperature constant at 80°C, we aimed to isolate the influence of drying time alone. Additionally, starting with this fixed temperature allows for a direct comparison with some results reported by Kumar et al., (2014).

**FIGURE 2:**
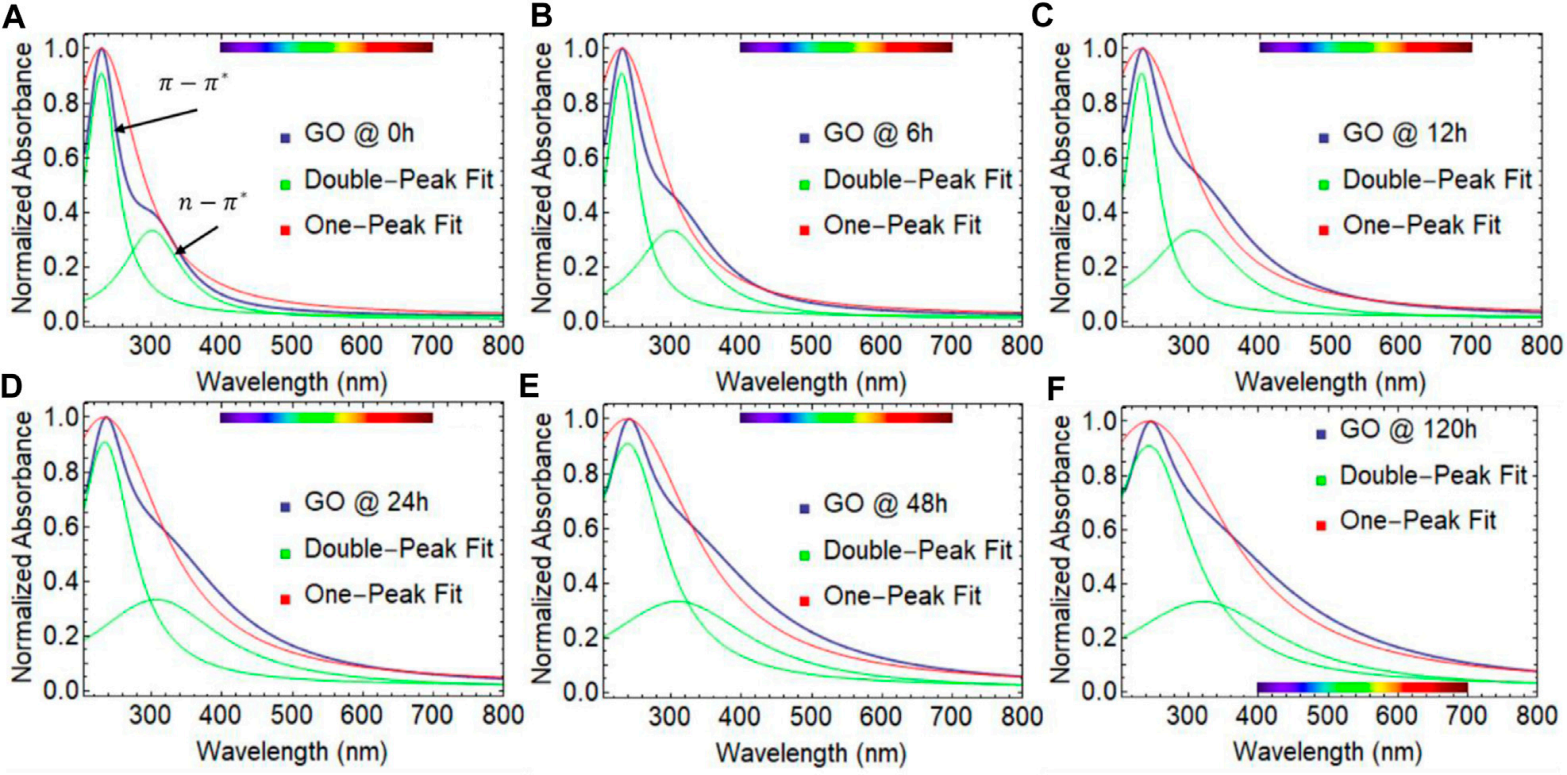
UV-visible spectra of graphene oxide (GO) dried at 80 ℃ considering different drying times: **(A)** 0 h, **(B)** 6 h, **(C)** 12 h, **(D)** 24 h, **(E)** 48 h, and **(F)** 120 h. The red and green curves represent the one- and double-peak Lorentz fit, respectively.

Figure 2 depicts the absorption spectra of GO at 80°C with varying sample drying times, ranging from 0 to 120 h. The red and green lines represent the Lorentzian fit using one or two peaks. Two distinct electronic transitions are observed: the $\pi -\pi^{*}$ transition and the $n-\pi^{*}$ transition (see Supplementary Figure S1 for energy levels). All the spectra are featureless in the visible region (400–700 nm) (Supplementary Figure S2). The wavelength position values of these electronic transitions can be found in Supplementary Table S1.

To contextualize, the $\pi -\pi^{*}$ transition is a type of electronic transition that occurs in molecules or materials containing conjugated π systems. A π system is a network of atoms with adjacent *p* orbitals that overlap to form delocalized π molecular orbitals. During the $\pi -\pi^{*}$ electronic transition, an electron in the π bonding molecular orbital is excited to the corresponding $\pi^{*}$ anti-bonding molecular orbital. This transition typically results in the absorption of light in the ultraviolet or visible range. In the case of GO, the $\pi -\pi^{*}$ transition is related to the delocalized π bonding network present in the graphene plane.

On the other hand, the $n-\pi^{*}$ electronic transition involves the excitation of an electron from a non-bonding, or lone pair, orbital ( n ) to the anti-bonding $\pi^{*}$ orbital. The $n-\pi^{*}$ transition typically also results in the absorption of light in the ultraviolet or visible range. In the case of GO, the $n-\pi^{*}$ transition is related to the oxygen-containing functional groups that are present on the graphene surface. The presence of functional groups in GO can induce changes in the electronic structure of the graphene lattice, including the opening of a bandgap, which can significantly alter the electronic and optical properties of the material.

**FIGURE 3:**
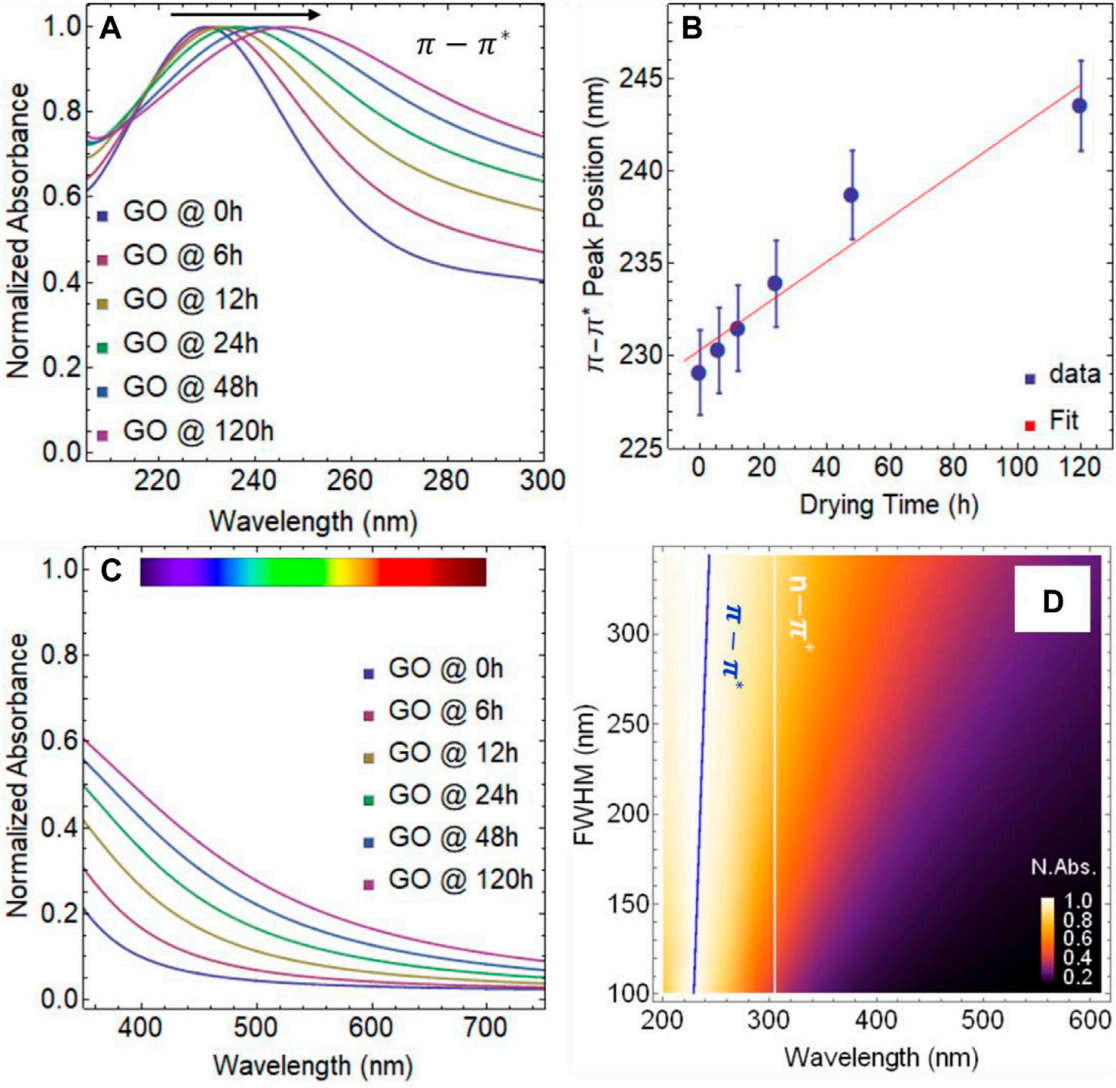
**(A)** UV-visible spectra of graphene oxide (GO) from 200 to 300 nm. **(B)** Position of the $\pi -\pi^{*}$ transition as a function of drying time. **(C)** UV-visible spectra of GO from 350 to 750 nm. **(D)** Normalized absorbance as a function of full-width half maximum (FWHM) *vs*. wavelength.

Then, one interesting observation is the shift in the position of the $\pi -\pi^{*}$ transition (Figure 3A), which changes from 229.63 nm after 0 h to 243.81 nm after 120 h. This shift corresponds to a change in energy from 5.40 to 5.09 eV. This observation shows that the electronic properties of the GO are changing over drying time, possibly due to the partial removal of oxygen-containing functional groups. Furthermore, we have analyzed the absorbance spectrum of GO at 144 h of drying (result not shown here), however, there is no significant disparity between the obtained result at 144 h ( $\lambda_{\pi -\pi^{*}}=244.13$ nm, $\lambda_{n-\pi^{*}}=311.9$ nm, FWHM = 342.11 nm, and R^2^ = 0.985) and the observed results at 120 h (Figure 2F). Given these observations, we focus our study on the drying window of 0–120 h and the one-peak fit approach through the text.

When performing a linear regression analysis on the data points of the $\pi -\pi^{*}$ transition shift over drying time (Figure 3B), the resulting equation is y=0.119 t+230.33 ( $R^{2}=0.929$ ). The slope of the line, 0.119, represents the rate of change of the $\pi -\pi^{*}$ transition shift concerning drying time. Specifically, a slope of 0.119 means that for every one-unit increase in time (e.g., 1 hour), the $\pi -\pi^{*}$ transition shift is expected to increase by 0.119 nm/h. This rate of change is constant and can be used to make predictions about the $\pi -\pi^{*}$ transition shift at future drying time points. However, it is important to note that there is a maximum shift detected for graphene dispersions, which is around 280 nm (Vacacela Gómez et al., 2021). This means that the value of y cannot continue to shift indefinitely over drying time, and will eventually reach a plateau, as starts to be observed at 120 (see Figure 3B). Additionally, the y -intercept of the line, 230.33 nm, represents the estimated initial position of the $\pi -\pi^{*}$ transition at time 0 h.

A marginal effect is observed in the position of the $n-\pi^{*}$ transition ( ∼ 305 nm), which suggests the presence of oxygenated functional groups, however, we point out that the structure of the absorbance spectrum is different at 120 h of drying time (Figure 2F). Interestingly enough, GO samples subject to continuous drying time at 80°C became strongly absorbent in the visible region (Figure 3C), showing an increase in the collection of photons in the wavelength range 350–700 nm.

As our study progresses, a previously unreported finding has come to light, a linear correlation between the full width at half maximum (FWHM) and absorbance spectra of dried GO samples. As the drying time increases, so does the width of the absorbance curve, which is constructed by both the $\pi -\pi^{*}$ and $n-\pi^{*}$ transitions. To illustrate this finding, Figure 3D displays the normalized absorbance as a function of FWHM vs. wavelength. A clear trend emerges as drying time increases, i.e., the spectral weight of FWHM increases from approximately 327 nm to almost 588 nm. As stated, the $\pi -\pi^{*}$ transition shifts towards longer wavelengths (blue line), while the $n-\pi^{*}$ transition remains relatively constant (white line).

### 3.2 Optical bandgap *vs*. drying time

In the Tauc approach, the absorption coefficient ( α ) is proportional to the energy of the incident photon energy ( E ) raised to the power of the Tauc exponent ( γ ), as given by the equation:

$${\left(\alpha h\nu \right)}^{1/\gamma }=B\left(h\nu -Eg\right)$$ (1)

where hν is the energy of the incident photons, Eg is the optical bandgap energy, and B is an energy-independent constant. Depending on nature transmission, γ can take the values of 1/2 for direct allowed transitions and 2 for indirect allowed transitions. In this sense, we assume an indirect bandgap nature as widely adopted (Romero et al., 2017).

**FIGURE 4:**
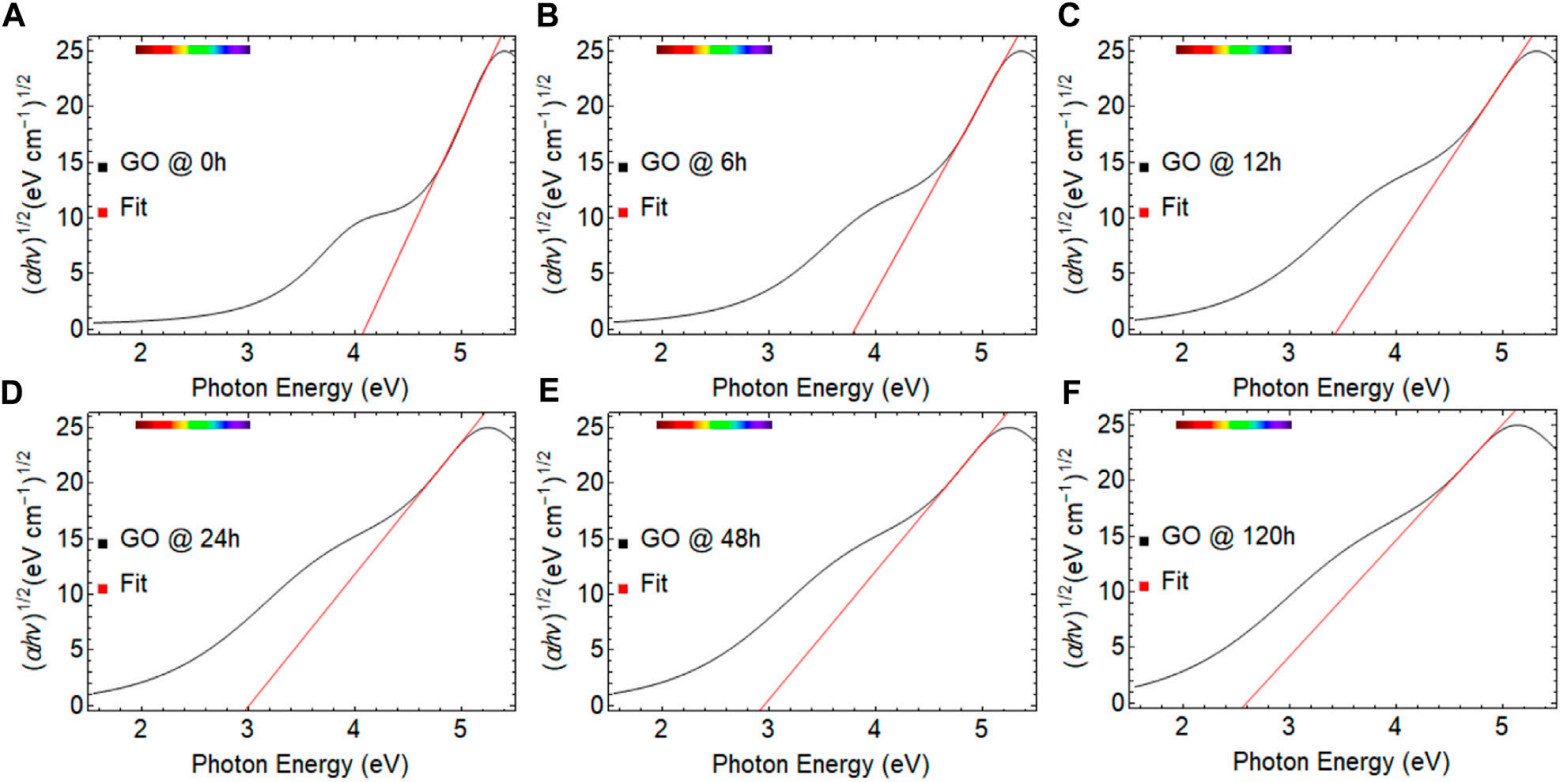
Tauc plots of graphene oxide (GO) dried at 80 ℃ considering different drying times: **(A)** 0 h, **(B)** 6 h, **(C)** 12 h, **(D)** 24 h, **(E)** 48 h, and **(F)** 120 h. The linear part of the plot is extrapolated to the x-axis (red line).

To determine the optical bandgap of GO using the Tauc approach, we first analyzed the absorbance spectrum of each sample. Next, we plotted the absorption coefficient ( α ) as a function of the photon energy ( hν ). Using this data, we generated a Tauc plot by plotting ${\left(\alpha h\nu \right)}^{1/2}$ on the y -axis and hν on the x -axis. By identifying the intercept of the linear section of the plot with the x -axis ( hν = 0), we estimated the optical bandgap of GO samples (Figure 4).

Figure 4 depicts the Tauc plot of GO at 80°C, where the drying time was varied from 0 to 120 h. The red line corresponds to the fitted region showing a steep linear increase of light absorption with increasing energy, which is a characteristic of semiconductor materials (Makuła et al., 2018). Table 1 provides the optical bandgap values estimated for each GO sample.

**TABLE 1:** Estimated optical bandgap values of graphene oxide (GO) as a function of drying time from 0 to 120 h. R^2^ is the coefficient of determination (R-squared).

Drying time (h)	Optical bandgap (eV)	R^2^
0	4.09	0.996
6	3.82	0.996
12	3.48	0.998
24	3.08	0.998
48	2.94	0.998
120	2.76	0.999

In Figure 4, all spectra exhibit a marked change in absorbance in the energy region of 4.1–5.5 eV, which is also characteristic of wide bandgap semiconductors. This pronounced change corresponds to the $\pi -\pi^{*}$ transition in GO in the wavelength region of 200–300 nm (Figure 2). Previous studies have estimated the bandgap by fitting the second linear region, which in our case is located between 3.0 and 4.0 eV and corresponds to the $n-\pi^{*}$ transition (300 and 400 nm, Figure 2). However, this approach is flawed and leads to an underestimation of the optical bandgap value. It is worth noting that the current study almost allows for the fitting of the second linear region after 120 h of drying time due to the displacement of the first linear region towards lower energy values (Figure 3F). An important aspect to consider is that with increasing drying time, the optical bandgap decreases from 4.09 eV (0 h) to 2.76 eV (120 h) (Table 2).

**TABLE 2:** Optical absorption coefficient estimated by a linear fit of the optical absorbance over cell length as a function of concentration under different drying times. R^2^ is the coefficient of determination (R-squared).

Material	Absorption coefficient (ml mg^-1^ m^-1^)	R^2^
GO @ 0 h	3932.22	0.992
GO @ 48 h	4586.71	0.992
GO @ 120 h	5507.15	0.985

**FIGURE 5:**
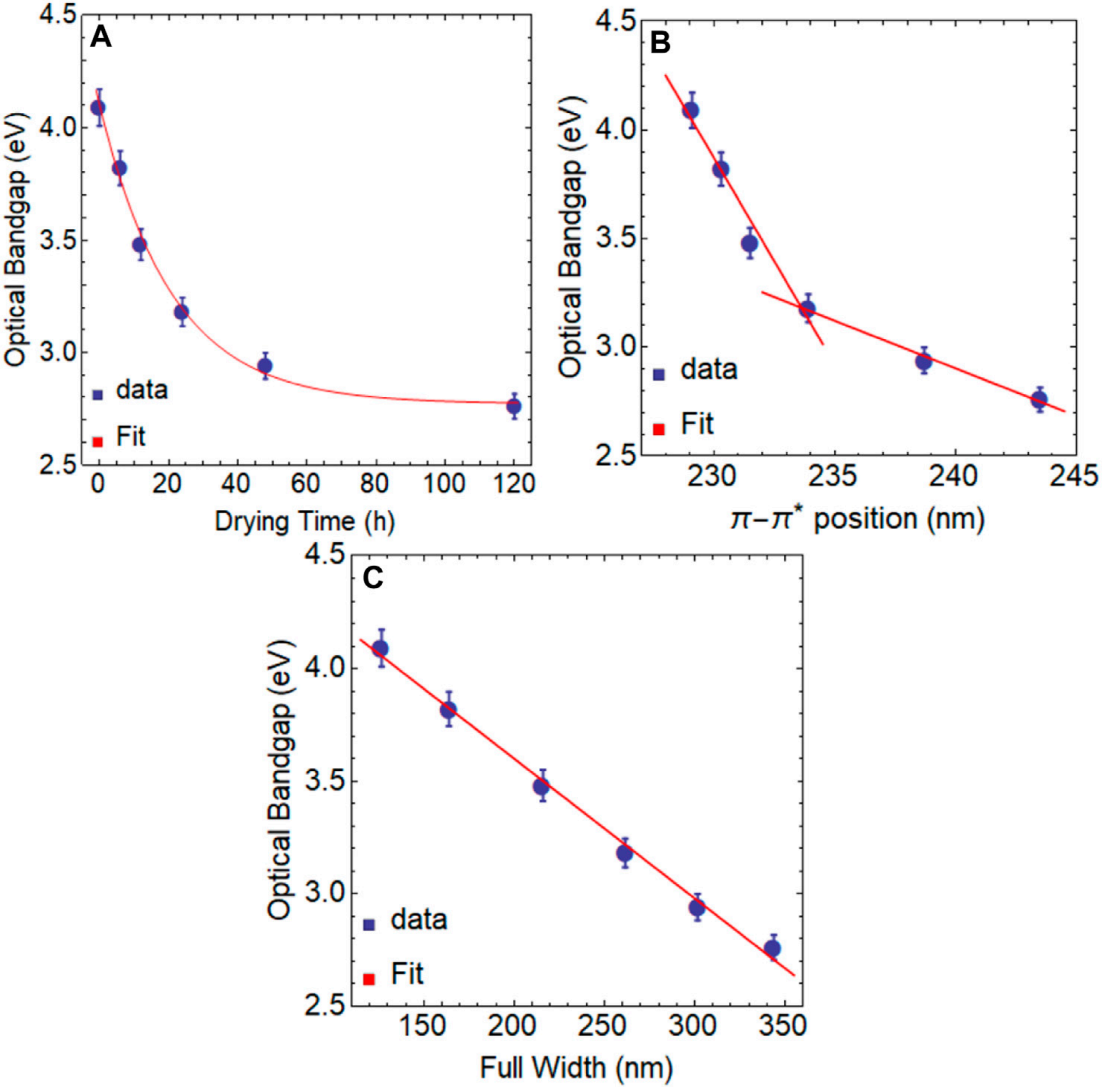
Optical bandgap of graphene oxide (GO) as a function of **(A)** drying time, **(B)** position of the $\pi -\pi^{*}$ transition, and **(C)** full width at half maximum (FWHM).

In Figure 5A, we present the relationship between the optical bandgap and the drying time, which exhibits a clear exponential decreasing trend. By performing a fit analysis, we obtained the expression: $y=1.33\;e^{-0.048\;t}+2.77.$ While we observe a negative decreasing rate in the relationship between the optical bandgap and the drying time, the rate of change is relatively small ( $-4.78\times 10^{-2}$ /h). Specifically, for drying times up to 120 h, we observe a decrease of approximately 1.33 eV in the optical bandgap. Moreover, this equation demonstrates that it is not feasible to extend its application to a hypothetical optical bandgap of y=0 eV, which corresponds to zero-gap graphene. This is because the maximum attainable optical bandgap is 2.77 eV by using the drying time effect. In a real situation, achieving full recovery of the graphene structure may not be feasible due to the presence of embedded oxygen functional groups in its basal plane that are challenging to remove.

It is important to mention that materials possessing a bandgap of ∼ 3 eV are highly versatile and can be utilized in numerous fields. Wide bandgap materials are ideal for use in the top layer of multi-junction solar cells, high-speed electronics, UV photodetectors, and optoelectronic devices. Wide bandgap materials are also used in the creation of blue or violet LEDs and lasers, which have numerous applications in lighting, displays, and optical communications.

Moreover, we have uncovered an inverse correlation between the position of the $\pi -\pi^{*}$ transition and the optical bandgap, which exhibits two regions of linearity (Figure 5B). The first is observed between 230 nm and 234 nm, while the second lies between 235 nm and 245 nm. Our findings demonstrate that as the $\pi -\pi^{*}$ transition shifts toward the red end of the spectrum, the bandgap decreases, leading to the restoration of the graphene properties. As well, we have observed a stronger linear relationship between the optical bandgap and FWHM (Figure 5C), indicating that as the bandgap decreases, the width of the absorption curve should increase proportionally.

### 3.3 Absorption coefficient *vs*. drying time

**FIGURE 6:**
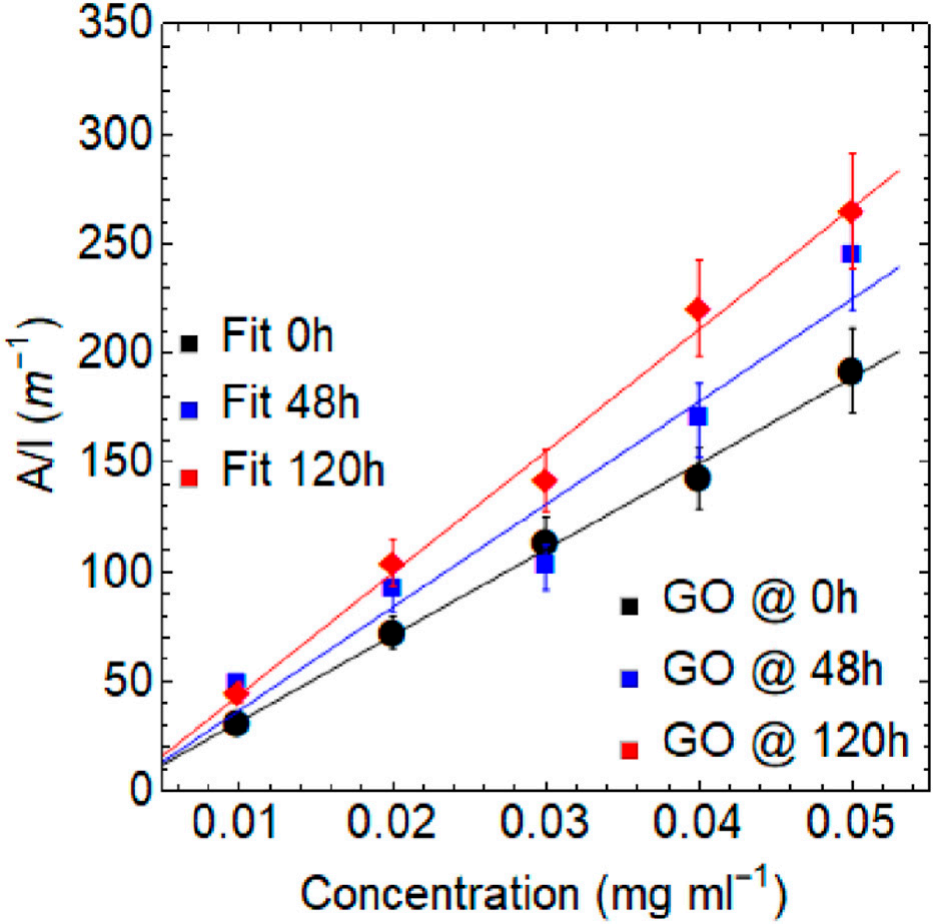
Optical absorbance at 660 nm divided by cell length as a function of concentration for graphene oxide (GO) subject to different drying times (0, 48, and 120 h).

The adsorption coefficient ( α ) was calculated according to the well-known expression of the Beer-Lamber law:

$$A=\alpha_{660}\;c\;l$$ (2)

where A is the absorbance data, c is the concentration, and l is the cuvette path length. The value of α was calculated by preparing a series of dispersions at given concentrations. As shown in Table 2; Figure 6, the value of α at 660 nm increases by increasing the drying time. For each given sample, the absorbance increases linearly with increasing concentration, indicating that GO dispersions follow the behavior of Eq. 2.

The values of α were determined by calculating the slope of the linear fit. The results show that α is approximately 3932 ml mg^−1^ m^−1^ at 0 h, 4587 ml mg^−1^ m^−1^ at 48 h, and 5507 ml mg^−1^ m^−1^ at 120 h. It is worth noting that these absorption coefficients are similar in orders of magnitude to the reported value for exfoliated graphene dispersions in water or alcohols, which is around 2460 ml mg^−1^ m^−1^, with a $\pi -\pi^{*}$ transition observed at approximately 265 nm (Hernandez et al., 2008).

The absorption coefficient of GO is higher than that of exfoliated graphene, likely due to the presence of oxygen-containing functional groups on its surface. These functional groups introduce defects in the hexagonal carbon lattice of graphene, creating localized states that can interact with photons at a wider range of energies than the delocalized π− electrons in pure graphene. This interaction results in a higher absorption coefficient for GO. In addition, the oxygen functional groups on the surface of GO can induce dipole moments and charge transfer, further enhancing the absorption of electromagnetic radiation. Therefore, the higher absorption coefficient of GO compared to graphene makes it useful for applications such as photovoltaics, photocatalysis, and optoelectronics.

### 3.4 Effect of temperature

The impact of temperature on the optical properties of GO is examined in this section. Four different temperatures, namely 40°C, 60°C, 80°C, and 100°C, have been selected for the experiment. The duration of the experiment has been set at 24 h to ensure homogeneity, as the water in the GO dispersion evaporates rapidly at 100°C. Therefore, a fixed duration of 24 h has been chosen to keep the experimental conditions as consistent as possible.

**FIGURE 7:**
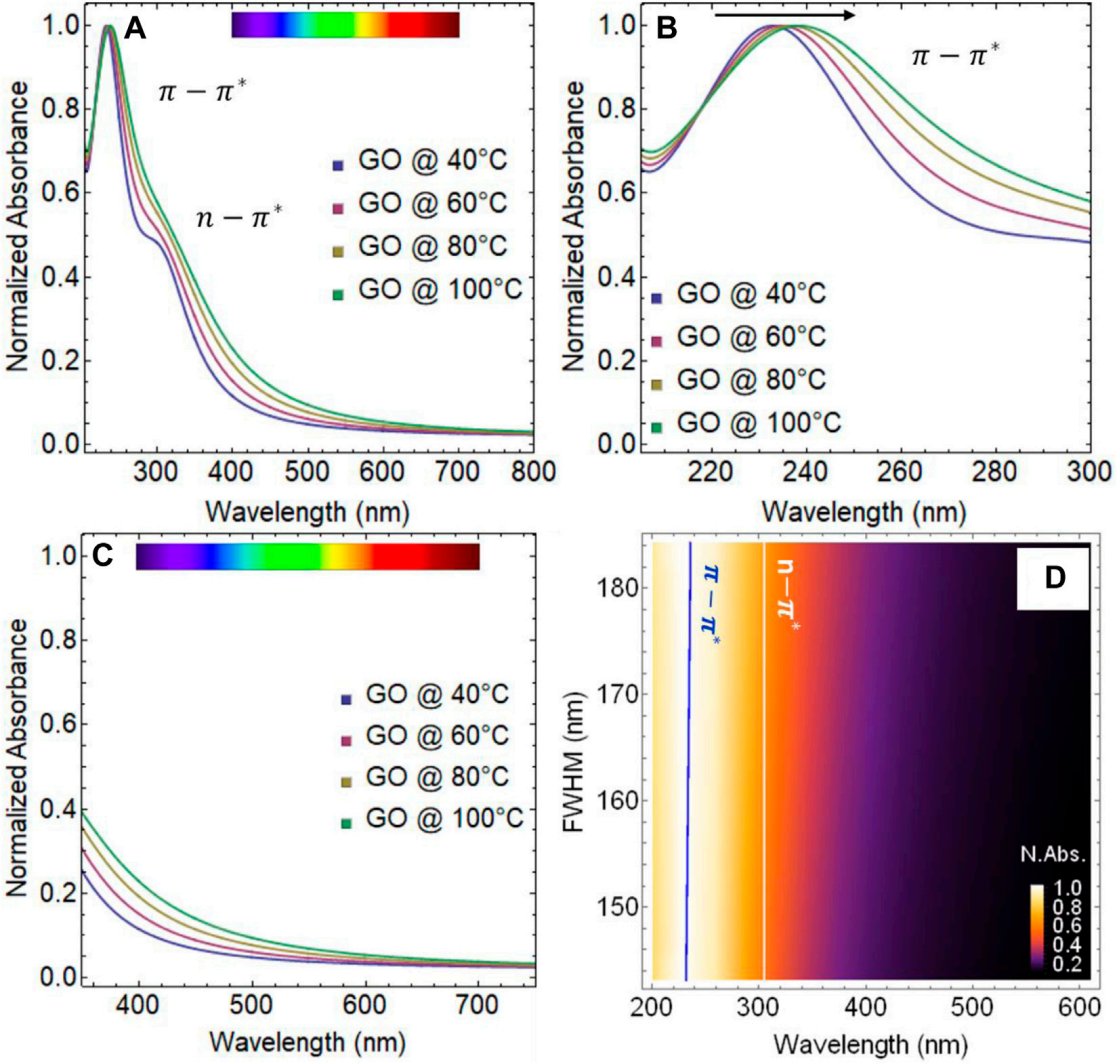
UV-visible spectra of graphene oxide (GO) **(A)** from 200 to 800 nm, **(B)** from 200 to 300 nm, **(C)** from 350 to 750 nm. **(D)** Normalized absorbance as a function of full-width half maximum (FWHM) *vs*. wavelength.

The absorbance spectra of GO subjected to different temperatures are shown in Figure 7A and the corresponding position of the $\pi -\pi^{*}$ and $n-\pi^{*}$ transitions can be found in Supplementary Table S2. It appears that the effect of temperature for 24 h is minimal. The absorbance spectrum shows visible $\pi -\pi^{*}$ and $n-\pi^{*}$ transitions and no significant change in their structure are observed with varying temperatures (Supplementary Figure S3). Even at 40°C and 100°C, the $\pi -\pi^{*}$ transition is located at about 231 and 236 nm, respectively (Figure 7B), indicating a shift of less than 6 nm. Moreover, the effect of temperature on the $n-\pi^{*}$ transition is also found to be marginal, and it remains relatively constant at ∼ 301 nm. As well, GO samples subject to different temperatures became also good absorbent in the visible region (Figure 7C), in the wavelength range 350–700 nm, but this effect is less than that observed under drying time (Figure 3C).

Figure 7D displays the normalized absorbance as a function of FWHM vs. wavelength. The blue line indicates that the shift of the $\pi -\pi^{*}$ transition is minimal, while the white line shows that the $n-\pi^{*}$ transition remains constant. Building on our previous analysis, we can infer that temperature has a less significant impact on steepening the GO absorption spectrum curve compared to drying time, as evidenced by the less pronounced FWHM spectral weight from 371 to 420 nm.

**FIGURE 8:**
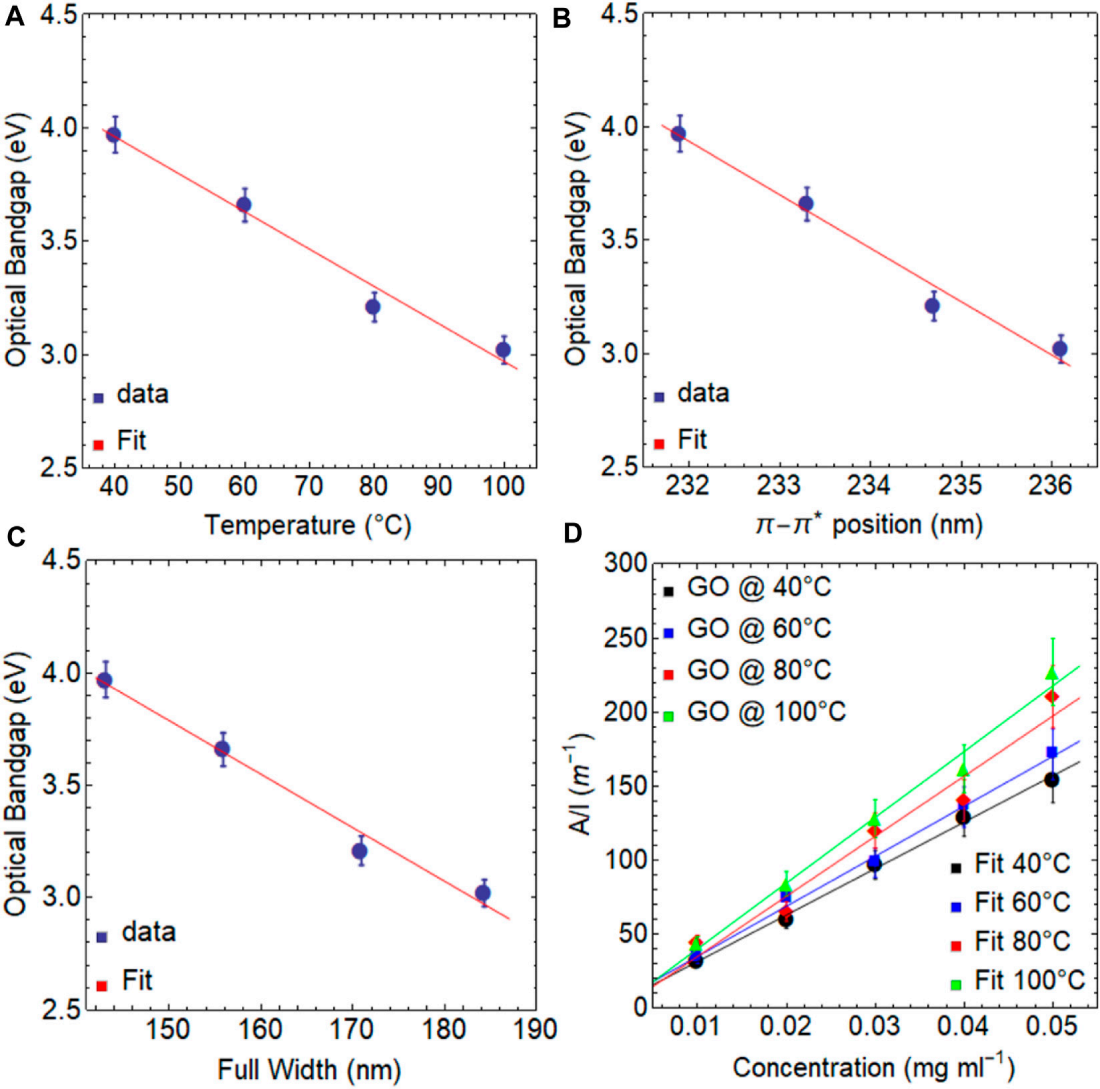
Optical bandgap of graphene oxide (GO) as a function of **(A)** drying time, **(B)** position of the $\pi -\pi^{*}$ transition, **(C)** full width at half maximum (FWHM), and **(D)** Optical absorbance as a function of concentration for graphene oxide (GO) subject to different temperatures.

Figure 8A displays a linear correlation between the optical bandgap and temperature. The optical bandgap decreases from 3.97 eV at 40°C to 3.06 eV at 100°C (Table 3, Supplementary Figure S4), with a variation of less than 1.0 eV. A linear fit of these data yields the following expression: $y=-1.65\times 10^{-2}\;t+4.62$ , indicating that a temperature of 280°C is required to reach a bandgap of zero (freestanding graphene). However, the reduction process of GO is complex and often necessitates higher temperatures or strong chemical reductant agents, as mentioned at the beginning of Section 3. Then, as seen in Figure 5A, the bandgap tends to follow a decreasing exponential trend due to achieving full recovery of the graphene properties is a challenge.

**TABLE 3:** Estimated optical bandgap values of graphene oxide (GO) as a function of temperature from 40 to 100 ℃ . R^2^ is the coefficient of determination (R-squared).

Temperature ( ℃ )	Optical bandgap (eV)	R^2^
40	3.97	0.998
60	3.70	0.996
80	3.19	0.997
100	3.06	0.998

As well, a linear correlation is also observed between the optical bandgap and the position of the $\pi -\pi^{*}$ transition (Figure 8B) or FWHM (Figure 8C).

Figure 8C; Table 4 demonstrate a trend similar to what was discussed previously. As the temperature increases, the absorption coefficient increases from 3140 ml mg^−1^m^−1^ at 40°C to 4061 ml mg^−1^ m^−1^ at 100°C. However, these values are slightly lower than those obtained due to the effect of drying time (see Table 2). All these results demonstrate the remarkable versatility of graphene oxide in modulating its electronic transitions, optical bandgap, and absorption coefficient through simple control of temperature and drying time.

**TABLE 4:** Optical absorption coefficient estimated by a linear fit of the optical absorbance over cell length as a function of concentration under different temperatures. R^2^ is the coefficient of determination (R-squared).

Material	Absorption coefficient (ml mg^-1^ m^-1^)	R^2^
GO @ 40 ℃	3139.97	0.997
GO @ 60 ℃	3382.52	0.996
GO @ 80 ℃	4061.18	0.962
GO @ 100 ℃	4443.84	0.988

### 3.5 Elemental composition and surface morphology

EDS analysis is a powerful technique that can be used to investigate the elemental composition of GO. Specifically, it can identify the presence of elements such as carbon, oxygen, and impurities. In this study, a sufficiently large area of GO samples was investigated using EDS to ensure the reliability of the results. However, it is important to note that to obtain a complete characterization of the material, other analytical techniques may also need to be employed in conjunction with EDS.

**FIGURE 9:**
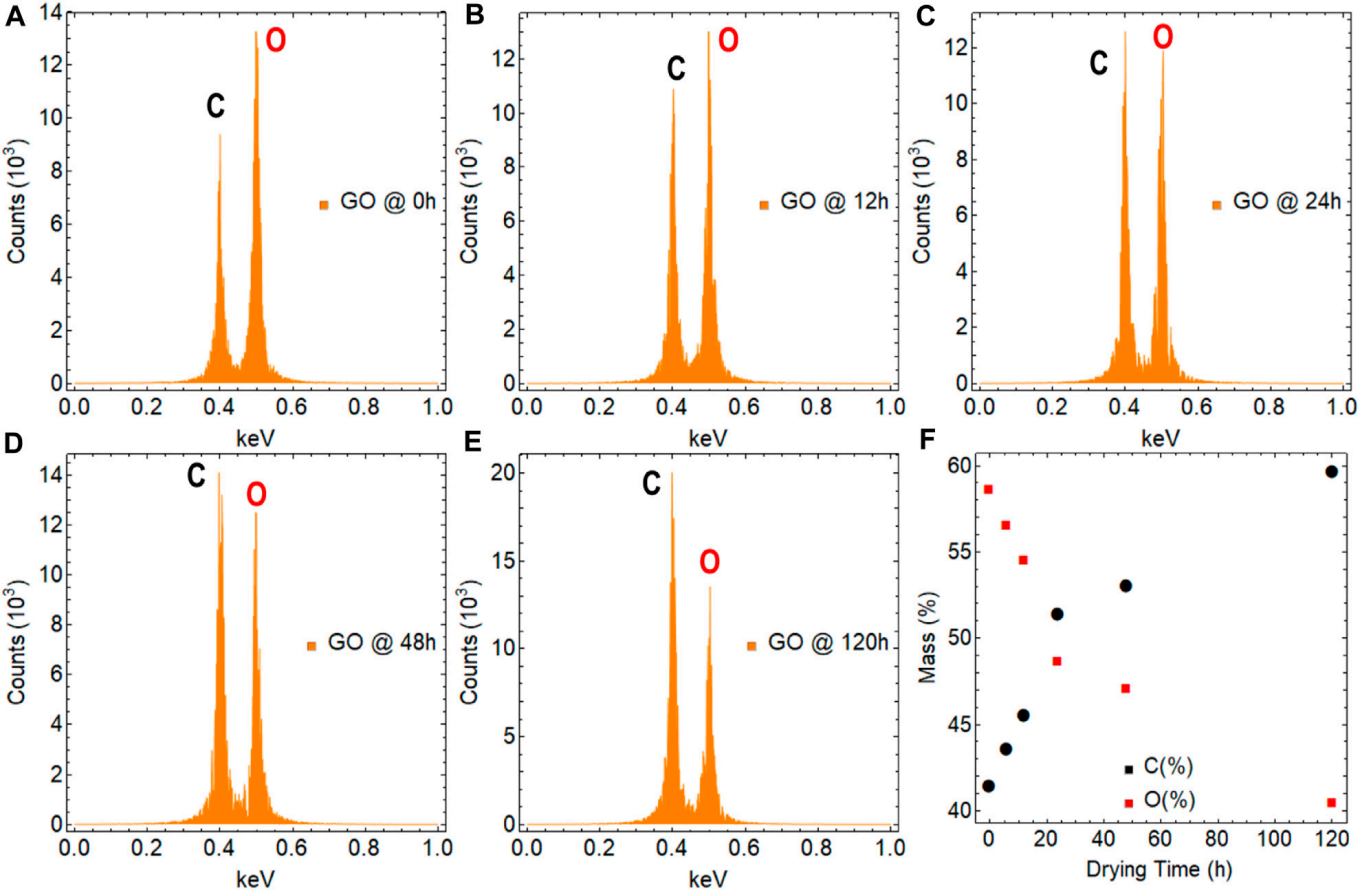
EDS measurements on graphene oxide (GO) subjects at different temperatures: **(A–E)** 0–120 h while maintaining a fixed temperature of 80°C, respectively. **(F)** Percentual mass as a function of drying time.

The results obtained are presented in Figure 9; Supplementary Figure S6, and Supplementary Table S3. To provide a basis for comparison, we first analyzed graphite (Supplementary Figure S5), which is composed primarily of carbon. As expected, the analysis showed a high carbon content of approximately 99%, which is consistent with the elemental composition of natural graphite. While this result is not surprising, it serves as a useful reference point for comparing the results obtained from other samples that may have more complex elemental compositions such as GO under the effect of drying time and temperature.

The impact of drying time on the oxygen content of GO can be understood by analyzing samples at two extreme points, at 0 h (Figure 9A) and 120 h (Figure 9E). The results indicate that as drying time increases, the oxygen content decreases from approximately 59%–40% (Figure 9F), indicating the removal of oxygen in various functional groups. This reduction in oxygen content, mainly, is attributed to the evaporation of water and other oxygen-containing functional groups (Saxena et al., 2011). These findings have implications for understanding the stability and properties of GO and can inform strategies for optimizing its synthesis and postprocessing conditions.

As well, the influence of temperature on GO can be examined by investigating extreme values, specifically at 40°C and 100°C. Intriguingly, exposing GO to 40°C for 24 h led to a rise of around 64% in oxygen content. This effect can be attributed to the fact that the resulting GO sample remained in the form of an aqueous dispersion after 24 h, and the evaporated water molecules could become intercalated within the internal structure of GO. Conversely, after 24 h at 100°C, the oxygen content decreased to 43%. This can be attributed to the fact that even though the sample was dry, the water molecules that were trapped and intercalated within GO required more time to escape from the internal structure.

**FIGURE 10:**
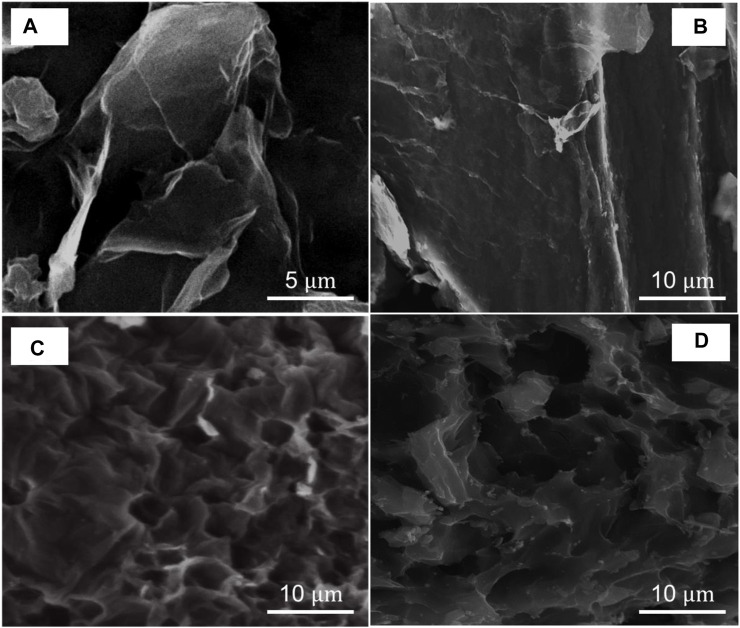
SEM images of graphene oxide (GO) under different drying times while maintaining a fixed temperature of 80°C: **(A)** 0 h and **(B)** 120 h as well as under different temperatures for 24 h of drying: **(C)** 40 ℃ and **(D)** 100 ℃ .

The morphology of the samples is illustrated in Figure 10. The starting graphite powder used in this study exhibited medium to large, corrugated flakes on the surface (Supplementary Figure S7). Conversely, the GO sample with 0 h of drying time exhibited a relatively smaller size, surface wrinkles, and several folds on the edges, confirming the chemical exfoliation of the materials by oxidation (Figure 10A). Furthermore, GO subjected to 120 h of drying time displayed a similar behavior with folded edges, but its surface morphology is more uniform, implying the partial recovery of sp^2^ hybridization, at least at the base of the GO plane (Figure 10B).

Conversely, when GO was exposed to temperatures of 40°C (Figure 10C) and 100°C (Figure 10D) for 24 h, no discernible changes were observed, and the surface remained corrugated with several folded edges. This outcome suggests that GO continued to exhibit a predominant sp^3^ hybridization, which is attributed to the presence of oxygen functional groups that still existed in the GO structure.

The Raman analysis of GO under different conditions (i.e., drying time and temperature) (Supplementary Figure S8; Supplementary Table S4) reveals four prominent peaks: D, D**, G, and D′. Each peak represents specific molecular vibrations and provides insights into its structure (Arias Arias et al., 2020). The D peak ( ∼ 1341 cm^−1^) indicates lattice defects caused by the introduction of oxygen functional groups during oxidation. The D** peak ( ∼ 1480 cm^−1^) arises from double resonance Raman scattering, reflecting the density of electronic states and structural disorder. The G peak ( ∼ 1570 cm^−1^) corresponds to graphitic sp^2^ carbon domains, indicating the presence of graphene-like regions. The D′ peak ( ∼ 1690 cm^−1^) originates from sp^3^ carbon atoms due to epoxy or hydroxyl functional groups, reflecting their abundance. An important finding obtained from the Raman measurements is that the I_D_/I_G_ intensity ratio increases when the samples are subjected to drying, regardless of the drying time or temperature. For instance, the I_D_/I_G_ intensity ratio in GO goes from 1.07 at 0 h and 80°C to 1.33 after drying for 24 h at 100°C. These findings have been extensively discussed in our previous work (Arias Arias et al., 2020).

The EDS, Raman, and SEM results provided further confirmation that when operating at low temperatures ( ≤ 100°C), the drying time was the most crucial factor, which corroborates the earlier findings obtained from UV-visible spectroscopy and Tauc analysis.

**FIGURE 11:**
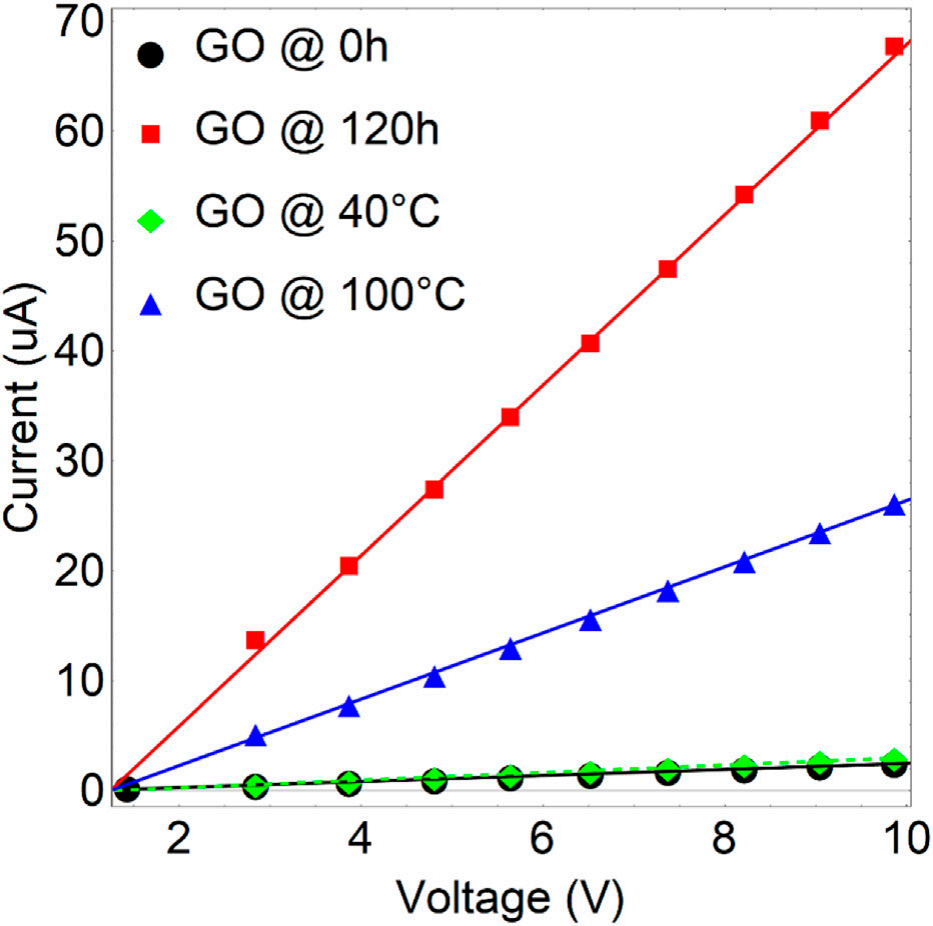
I-V characteristics of graphene oxide (GO) under varying drying times and temperatures are presented. The green and blue curves depict GO dried for 24 h at 40°C and 100°C, respectively. Meanwhile, the black and red curves represent GO dried at 80°C for 0 and 120 h, respectively.

To further characterize the treated samples, Figure 11; Supplementary Table S5 show the current vs. voltage plots (*I-V* curves) for GO treated at 0 h (black line), 120 h (red line), 40°C (green line), and 100°C (blue line). GO at 0 h ( $3.65\times 10^{6}\;\Omega$ ) and GO at 40°C ( $2.90\times 10^{6}\;\Omega$ ) exhibit insulating properties irrespective of the magnitude of the applied voltage. The current of GO at 120 h ( $1.29\times 10^{5}\;\Omega$ ) and GO at 100°C ( $3.32\times 10^{5}\;\Omega$ ) increase gradually with biasing the voltage, and the insulating characteristic starts to be modified. At 10 eV, the conductivity is around 2.5 (0 h), 67.5 (120 h), 3.0 (40°C), and 26.3 (100°C) μA . It has been observed that the resistance of GO is lowest after 120 h of drying time. This can be attributed to the removal of some oxygen functional groups. However, GO still contains numerous oxygen functional groups, which makes it an insulating material. This is evidenced and confirmed by the wide bandgap estimated through the Tauc approach (2.8 eV).

## 4 Conclusion

In summary, we explored the physical and chemical properties of GO treated at low temperatures. Our focus is on investigating the electronic and optical characteristics of GO and the changes in these features upon drying time (from 0 h to 120 h while maintaining a fixed temperature of 80°C) and low temperatures (from 40 ℃ to 100 ℃ with a constant drying time of 24 h).

We found that the $\pi -\pi^{*}$ transition is the most affected, shifting from approximately 230 nm–244 nm after 120 h of drying time, while the $n-\pi^{*}$ transition remains unchanged in wavelength but decreases in intensity. The absorption coefficient was measured to be 5507 ml mg^−1^ m^−1^ at 120 h, similar to the absorption coefficients in order of magnitude reported for dispersions of liquid-phase exfoliated graphene (2344 ml mg^−1^ m^−1^). The optical bandgap was found to be 2.8 eV for dried samples at 120 h. Furthermore, we found a linear relationship never noted between the optical bandgap and the position of the main absorbance peak or FWHM curve. The study is complemented by using EDS analysis, SEM measurements, and *I-V* curves. In particular, the EDS analysis revealed a notable trend as the drying time increased: the oxygen content decreased from approximately 59%–40%. This finding strongly suggests the removal of oxygen functional groups. Furthermore, SEM observations indicated distinct characteristics between GO samples with 0 h and 120 h of drying time. GO samples with 0 h of drying time exhibited smaller sizes, surface wrinkles, and multiple folds along the edges. Conversely, GO subjected to 120 h of drying time displayed a similar folded edge behavior, but its surface morphology appeared more uniform, indicating a partial recovery of sp^2^ hybridization. I-V measurements provided additional insights, showing that the resistance of GO was at its lowest ( $1.29\times 10^{5}\;\Omega$ ) after 120 h of drying time. The latter can be attributed to the effective removal of oxygen functional groups.

Our findings can be used to tailor the use of GO in various contexts by determining the optimal temperature and duration for a specific application. Additionally, this study provides valuable insights into the electronic transitions and optical properties of GO and fills a gap in the literature on low-temperature treatment processes for GO without strong reductant agents or controlled environments.

Finally, we would like to underscore the significance of our findings concerning the optical properties of GO, however, it is crucial to point out that a more comprehensive understanding of the results presented in this study could be attained through the inclusion of complementary XPS measurements. By integrating XPS analysis, one can be able to delve deeper into the intricate aspects of our findings and acquire a more thorough understanding of the observed phenomena.

## Data Availability

The authors declare that the data supporting the findings of this study are available within the paper (and its Supplementary Information files). Further raw data are also available from the corresponding author upon reasonable request.
