# Supplementary material for: Optical properties of graphene oxide
Source: Front Chem. 2023 Jul 20;11:1214072. doi: 10.3389/fchem.2023.1214072 (PMC10397392; doi:10.3389/fchem.2023.1214072)
Supplement: Supplementary file 1 [file DataSheet1.docx]

**SUPPLEMENTARY INFORMATION: Optical Properties of Graphene Oxide**

**Talia Tene^1^, Marco Guevara^2^, Freddy Benalcázar Palacios^3^, Tania Paulina Morocho Barrionuevo^4^, Cristian Vacacela Gomez^5^*, Stefano Bellucci^5^**

^1^ Department of Chemistry, Universidad Técnica Particular de Loja, Loja 110160, Ecuador

^2^ Facultad de Ingenieria Mecánica, Escuela Superior Politécnica de Chimborazo (ESPOCH), Riobamba 060155, Ecuador

^3^ Facultad de Ingeniería en Sistemas Electrónica e Industrial, Universidad Técnica de Ambato, Ambato 180104, Ecuador

^4^ Facultad de Ciencias, Carrera de Estadística, Escuela Superior Politécnica de Chimborazo (ESPOCH), Riobamba 060155, Ecuador

^5^ INFN-Laboratori Nazionali di Frascati, I-00044 Frascati, RM, Italy

* Correspondence:

Corresponding Author

[vacacela@lnf.infn.it](mailto:vacacela@lnf.infn.it)

bellucci@lnf.infn.it

**
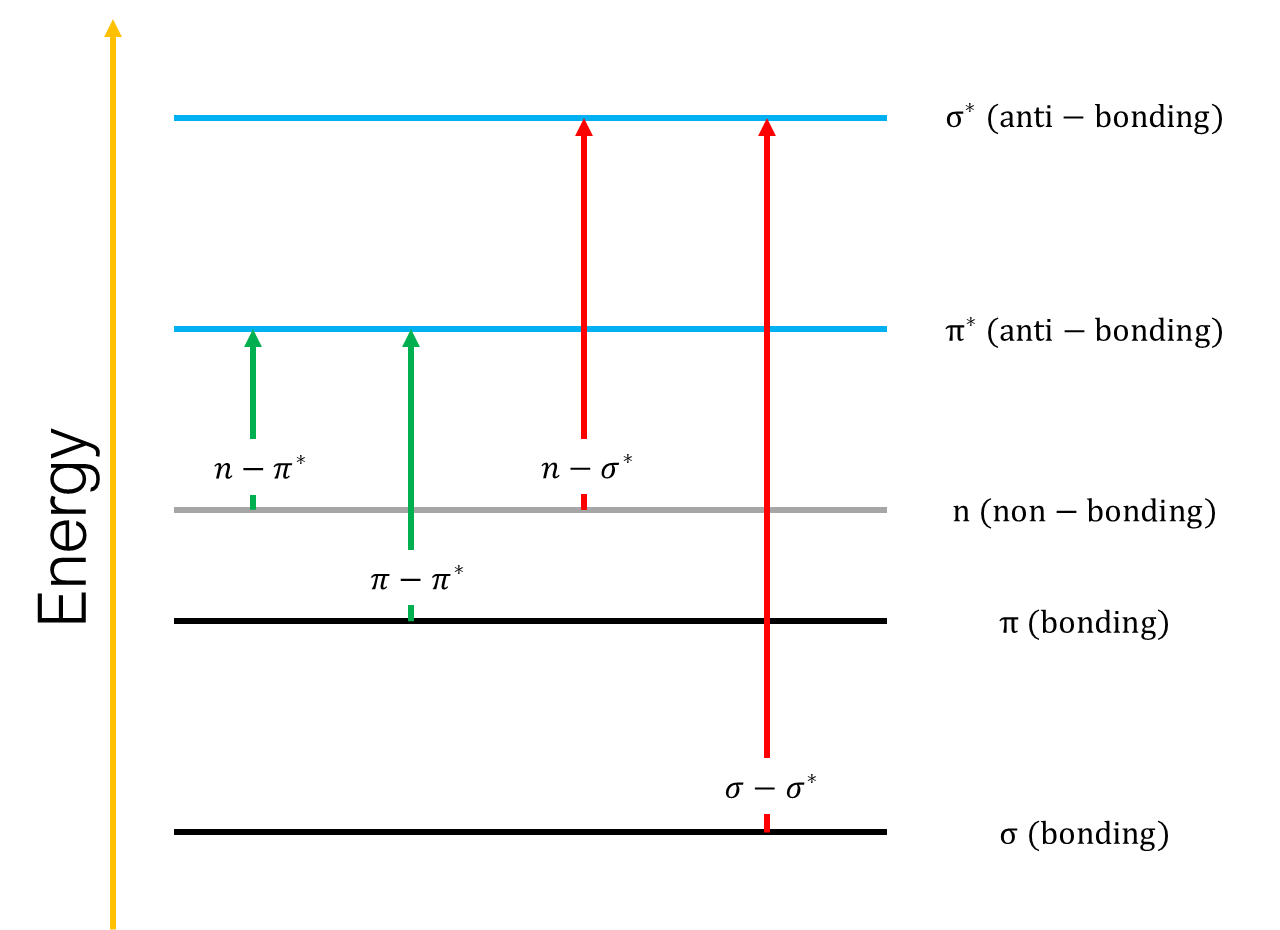
**

**Figure S1.** Molecular orbitals are illustrated at different energy levels.

**
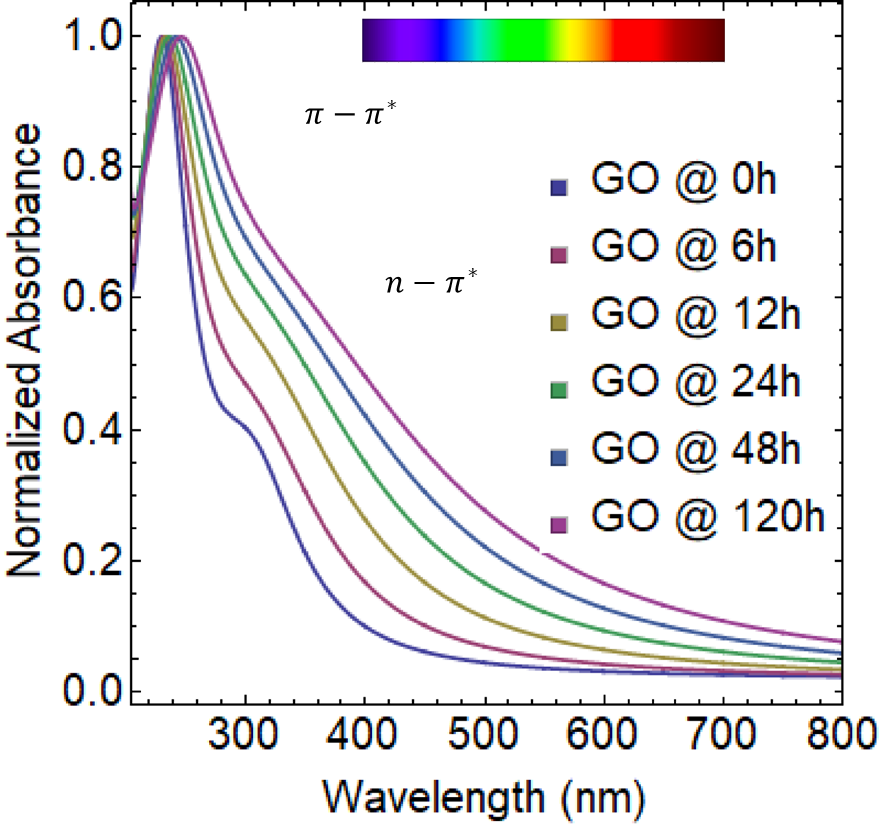
**

**Figure S2.** Full UV-visible spectra of graphene oxide (GO) subject to different drying times.

**
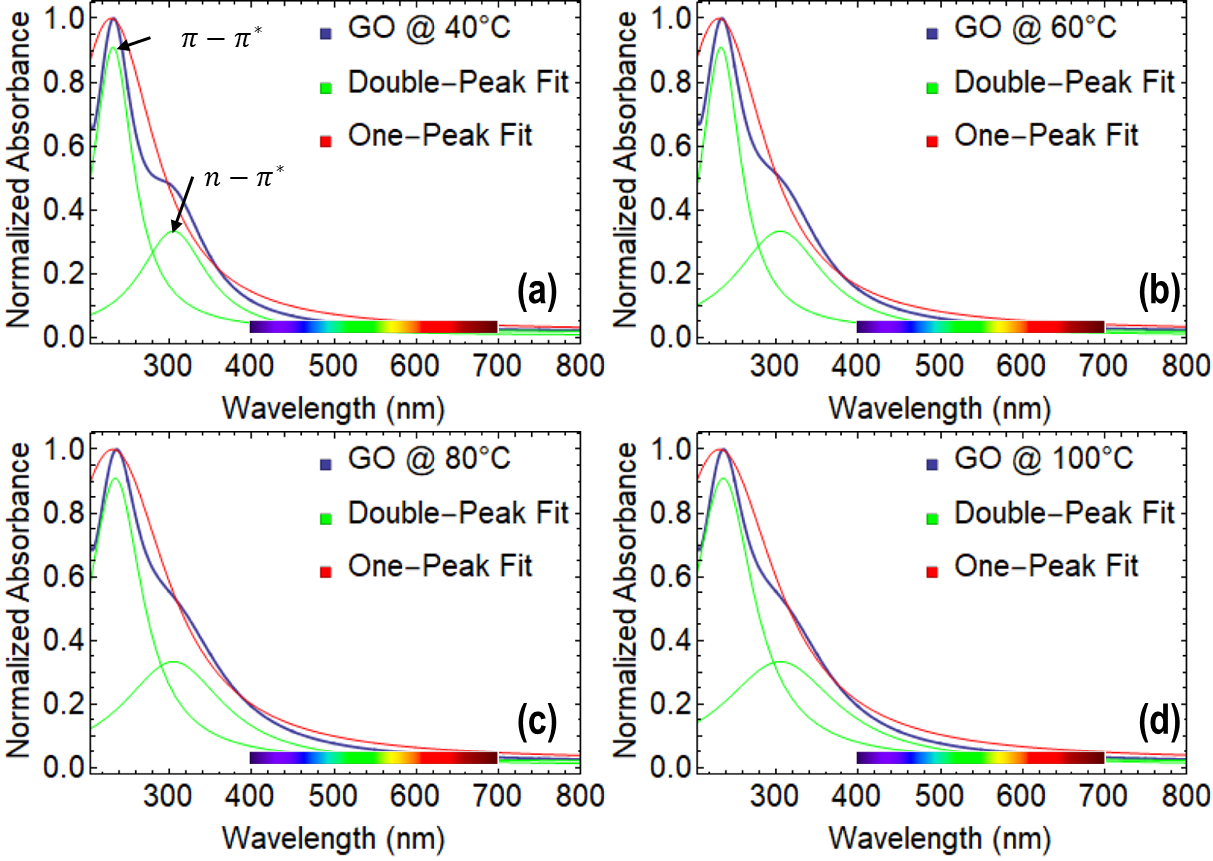
**

**Figure S3.** UV-visible spectra of graphene oxide (GO) dried for 24 h at different temperatures: (a) 40 $℃$, (b) 60 $℃$, (c) 80 $℃$, and (d) 100 $℃$.

**
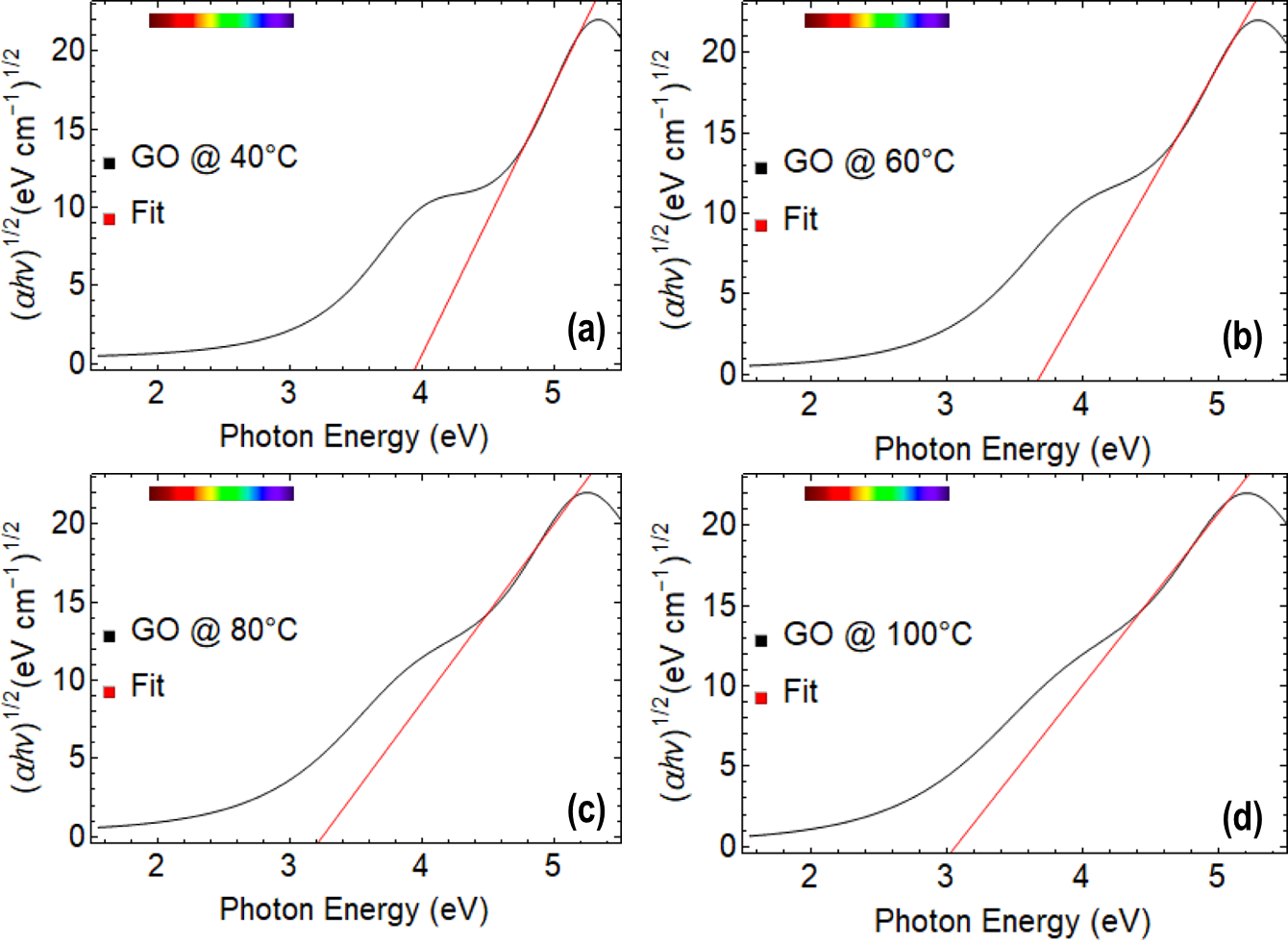
**

**Figure S4.** Tauc plots of graphene oxide (GO) dried for 24 h at different temperatures: (a) 40 ℃, (b) 60 ℃, (c) 80 ℃, and (d) 100 ℃. The linear part of the plot is extrapolated to the x-axis (red line).


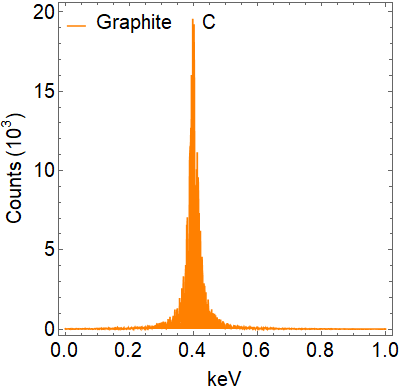


**Figure S5.** EDS measurements on graphite

**
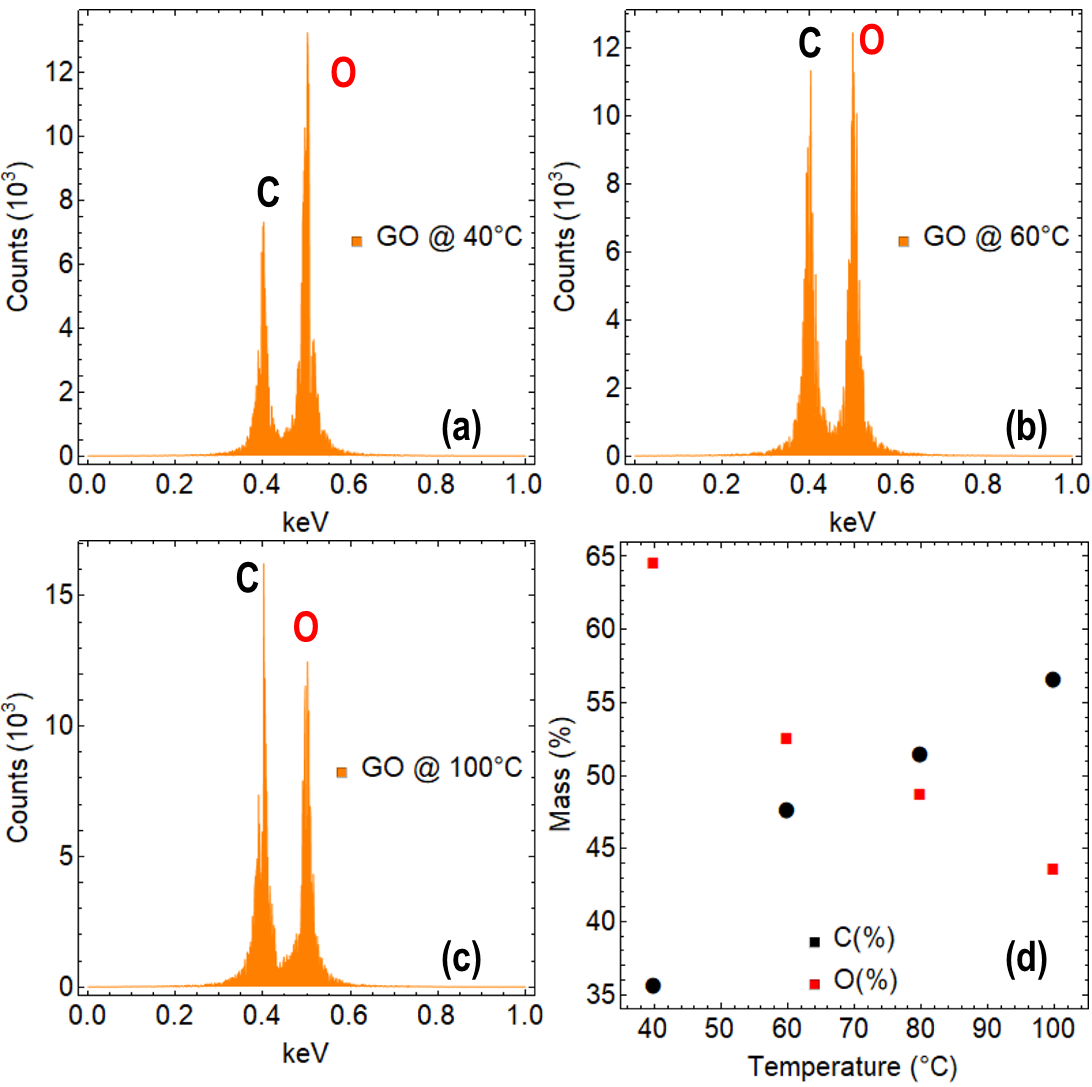
**

**Figure S6.** EDS measurements on graphene oxide (GO) with a constant drying time of 24 h at (a) 40 $℃$, (b) 60 $℃$, and (c) 100 $℃$. (d) Percentual mass as a function of different temperatures.

**
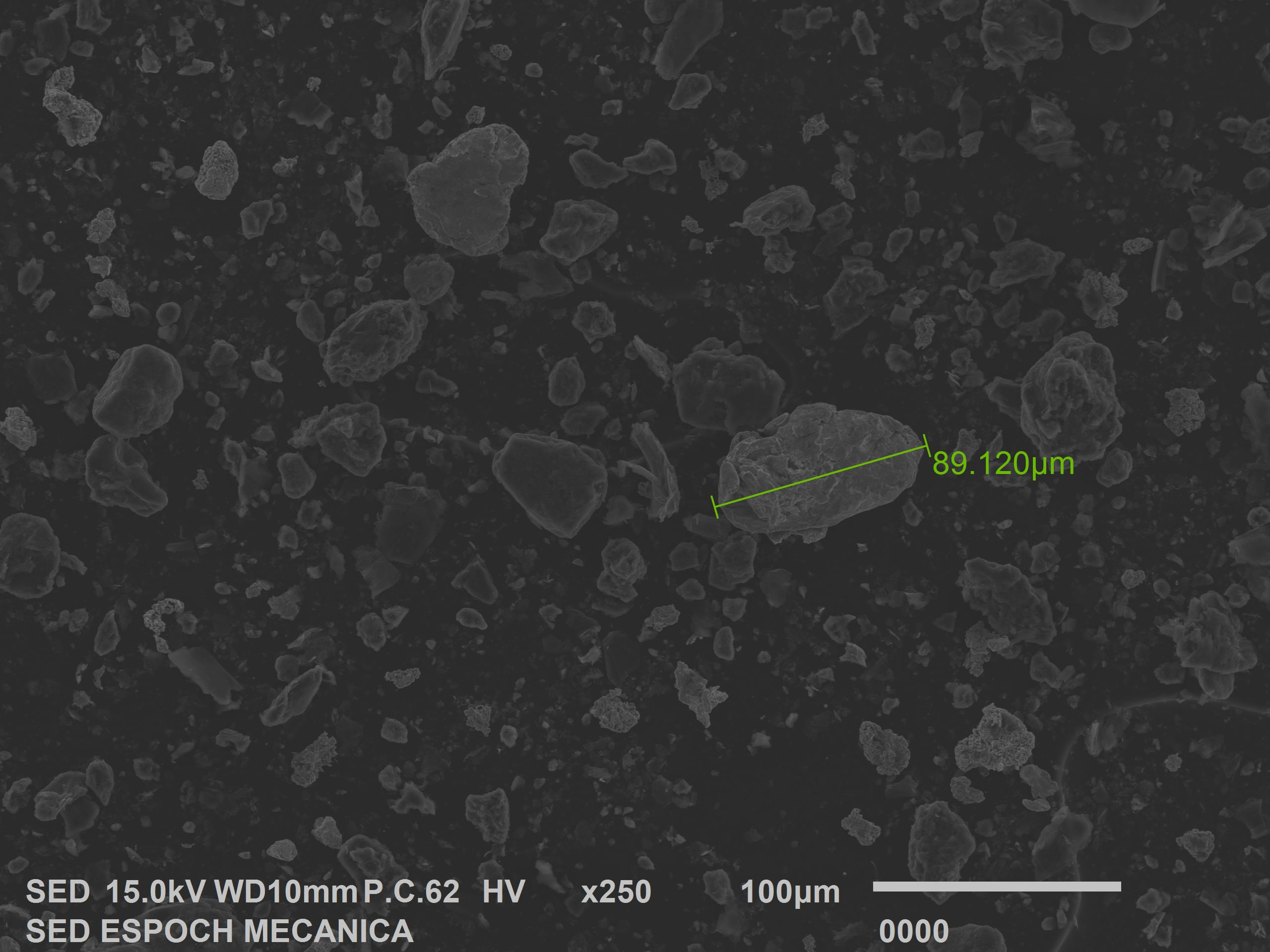
**

**Figure S7.** SEM morphology of graphite powder

**
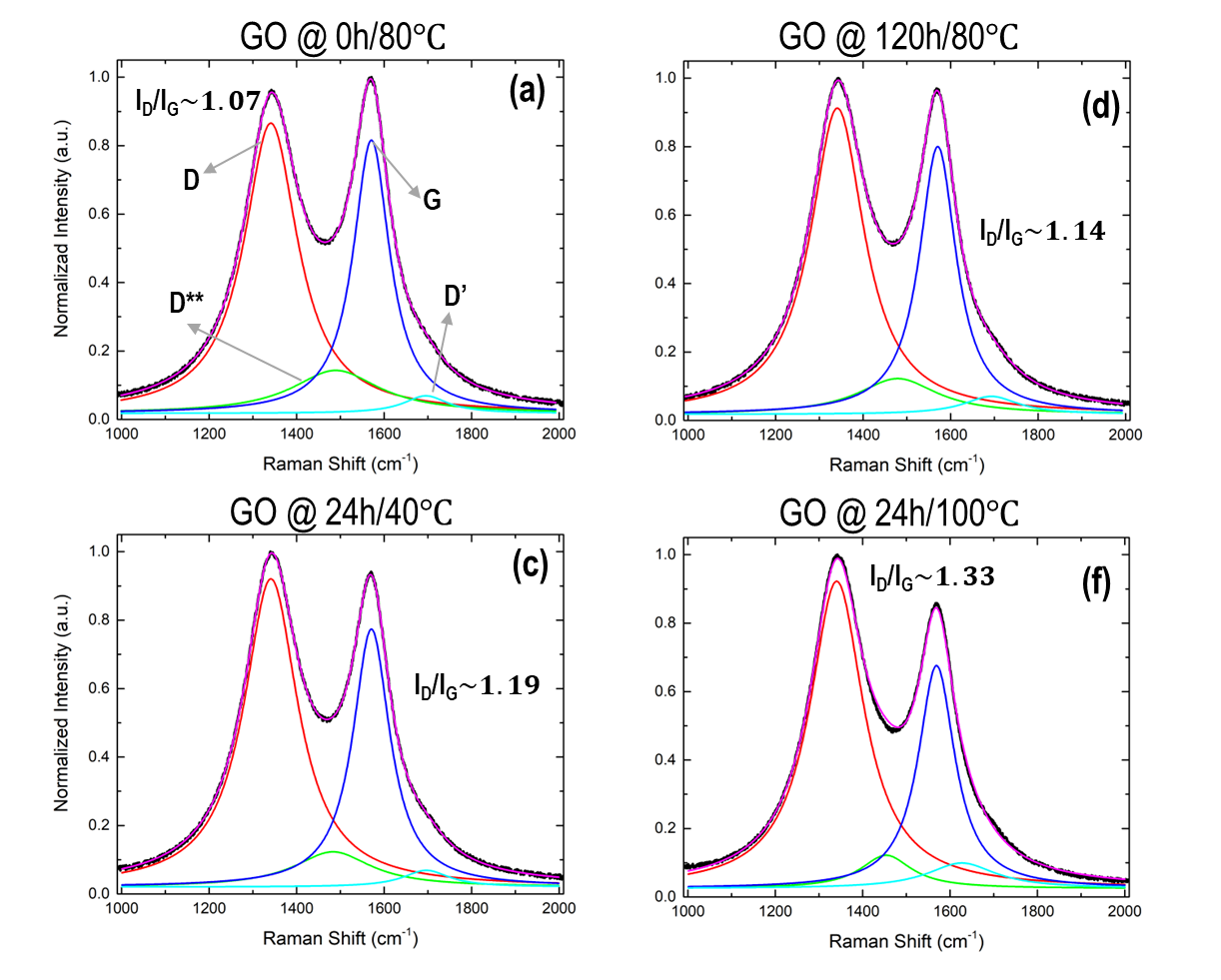
**

**Figure S8.** Raman analyzes in graphene oxide (GO) under different drying times while maintaining a fixed temperature of 80°C: (a) 0 h and (b) 120 h as well as under different temperatures for 24 h of drying: (c) 40 ℃ and (d) 100 ℃.

**Table S1.** Peak Position of $\pi-\pi^{*}$ and $n-\pi^{*}$transitions in graphene oxide (GO) and related full width at half maximums (FWHM), considering different drying times. R^2^ is the coefficient of determination (R-squared).

| **Drying Time (h)** | $\boldsymbol{\pi-}\boldsymbol{\pi}^{\boldsymbol{*}}$ **Transition (nm)** | $\boldsymbol{n-}\boldsymbol{\pi}^{\boldsymbol{*}}$ **Transition (nm)** | **FWHM (nm)** | **R^2^** |
| --- | --- | --- | --- | --- |
| 0 | 229.63 | 301.15 | 126.57 | 0.999 |
| 6 | 230.59 | 302.15 | 163.92 | 0.997 |
| 12 | 231.51 | 305.16 | 215.92 | 0.993 |
| 24 | 233.23 | 307.17 | 261.59 | 0.992 |
| 48 | 238.23 | 309.19 | 301.72 | 0.988 |
| 120 | 243.81 | 311.27 | 343.90 | 0.988 |

**Table S2.** Peak Position of $\pi-\pi^{*}$ and $n-\pi^{*}$ transitions in graphene oxide (GO) and related full width at half maximums (FWHM), considering different temperatures. R^2^ is the coefficient of determination (R-squared).

| **Temperature (^o^C)** | $\boldsymbol{\pi-}\boldsymbol{\pi}^{\boldsymbol{*}}$ **Transition (nm)** | $\boldsymbol{n-}\boldsymbol{\pi}^{\boldsymbol{*}}$ **Transition (nm)** | **FWHM (nm)** | **R^2^** |
| --- | --- | --- | --- | --- |
| 40 | 230.99 | 301.16 | 143.10 | 0.997 |
| 60 | 233.02 | 301.17 | 155.87 | 0.995 |
| 80 | 234.79 | 301.17 | 170.92 | 0.995 |
| 100 | 236.16 | 301.19 | 184.30 | 0.990 |

**Table S3.** Elemental composition of graphite and obtained GO samples. R^2^ is the coefficient of determination (R-squared).

| **Sample** | **C (%)** | **O (%)** |
| --- | --- | --- |
| Graphite | 99.1 | --- |
| GO @ 0 h | 41.47 | 58.53 |
| GO @ 6 h | 43.75 | 56.25 |
| GO @ 12 h | 45.56 | 54.44 |
| GO @ 24 h | 51.42 | 48.58 |
| GO @ 48 h | 53.01 | 46.99 |
| GO @ 120 h | 59.65 | 40.35 |
| GO @ 40 $℃$ | 35.60 | 64.40 |
| GO @ 60 $℃$ | 47.66 | 52.34 |
| GO @ 100 $℃$ | 56.56 | 43.44 |

**Table S4**. Raman-peak position of graphene oxide (GO) under different conditions. R^2^ is the coefficient of determination (R-squared).

| **Sample** | **D (cm^-1^)** | **D** (cm^-1^)** | **G (cm^-1^)** | **D’ (cm^-1^)** | **R^2^** |
| --- | --- | --- | --- | --- | --- |
| GO @ 0 h | 1341.04 | 1489.50 | 1570.93 | 1695.54 | 0.999 |
| GO @ 120 h | 1341.10 | 1478.99 | 1570.59 | 1693.04 | 0.999 |
| GO @ 40 $℃$ | 1341.38 | 1500.02 | 1570.68 | 1662.89 | 0.999 |
| GO @ 100 $℃$ | 1340.50 | 1453.56 | 1569.06 | 1626.84 | 0.999 |

**Table S5.** Resistance of low-temperature treated graphene oxide (GO) is estimated from the linear fit of *I-V* curves in Figure 9. R^2^ is the coefficient of determination (R-squared).

| **Sample** | **Resistance (**$\boldsymbol{\Omega}$**)** | **R^2^** |
| --- | --- | --- |
| GO @ 0 h | $3.65\times{10}^{6}$ | 0.995 |
| GO @ 120 h | $1.29\times{10}^{5}$ | 0.999 |
| GO @ 40 $℃$ | $2.90\times{10}^{6}$ | 0.999 |
| GO @ 100 $℃$ | $3.32\times{10}^{5}$ | 0.999 |
